# Equine Behavioural and Physiological Responses to Auditory Stimuli in the Presence and Absence of Noise-Damping Ear Covers

**DOI:** 10.3390/ani13091574

**Published:** 2023-05-08

**Authors:** Clare Hole, Rachel Murray, David Marlin, Paul Freeman

**Affiliations:** 1 Department of Veterinary Medicine, University of Cambridge, Cambridge CB3 0ES, UK; crmh4@cam.ac.uk (C.H.); pf266@cam.ac.uk (P.F.); 2 Rossdales Veterinary Surgeons, Cotton End Road, Exning, Newmarket, Suffolk CB8 7NN, UK; 3 AnimalWeb Ltd., Cambridge CB4 0WZ, UK; dm@davidmarlin.co.uk

**Keywords:** horse, auditory, behaviour, heart rate, sport performance

## Abstract

**Simple Summary:**

Auditory perception is a key driver in behavioural and physiological responses and as prey species, horses have evolved to identify these stimuli and respond appropriately to maximise chances of survival. Sport horses are required to perform without distraction by external, irrelevant auditory stimuli and to help achieve this, ‘noise-damping’ ear covers have been developed. This study investigated behavioural and physiological (heart rate) responses of horses to different sounds commonly present in a competition environment and compared these responses in the presence and absence of ear covers. A difference in both physiological and behavioural responses to different auditory stimuli was found, suggesting an ability to discriminate the sounds and alter responses based on the individual stimulus. An overall difference in both physiological and behavioural responses with and without ear covers was also found with a reduction in responsiveness when wearing ear covers, although responses varied between sounds presented. The attenuation of these responses when wearing ear covers implies a reduced perception of sounds with varying levels of effectiveness. We suggest this information can be used to predict responses of sport horses, enhancing management and optimisation of performance while improving horse welfare.

**Abstract:**

Despite numerous studies investigating responses to visual perception, there is limited research into how horses respond to different auditory stimuli. Although ‘noise-damping’ ear covers are frequently used on sport horses to minimise distraction from external auditory stimuli, the effectiveness of ear covers has not been established. This study aimed to (i) investigate the responses of horses to different sounds commonly present in a competition environment, and (ii) compare these responses in the presence and absence of ear covers. A total of 18 horses were presented with 5 sounds commonly heard in competition or stable environments both with and without ear covers, in a randomised order crossover design. Behavioural and heart rate responses were recorded. Responses were compared between sounds and with/without ear covers. Differences in physiological and behavioural responses to different complex auditory stimuli were shown. An overall difference in physiological and behavioural responses with and without ear covers was detected, although the only difference in heart rate between ear covers and no ear covers that individually achieved significance was the feed sound. These findings suggest that horses can discriminate sounds and alter their responses based on the individual stimulus, and these responses are reduced when wearing ear covers with varying effectiveness for different sounds.

## 1. Introduction

Auditory perception is central to the processing of sensory signals, which informs behavioural and physiological changes. Horses are known to be sensitive to auditory stimulation over a wide estimated range of approximately 55Hz–33.3kHz and can localise sound up to a threshold of 22° (the angle at which horses can correctly discriminate between two bursts of noise on 75% of trials) in order to detect and assess the identity of the sound source [1,2,3]. As prey species, horses have evolved mechanisms to identify the presence of a predator through natural selection, including the identification of auditory stimuli. Therefore, horses typically respond to potentially alarming stimuli by preparing for flight or by erratic movements. This physiological response is characterised by an increased heart rate through sympathetic stimulation via the autonomic nervous system, which is followed by the secretion of cortisol as part of a slower endocrine response. Thus, monitoring changes in heart rate can provide non-invasive physiological information not apparent in solely behavioural responses [4,5,6]. It has been suggested that behavioural features can act as indicators of the emotional state of horses, particularly when they are experiencing fear or anxiety as a response to novel stimuli [7,8]. There is therefore potential value to collecting both behavioural and physiological data to investigate both the autonomic response and more stimuli-specific behavioural reactions.

Both behavioural and physiological responses have been recorded widely in the past when examining responses to sensory stimuli, including visual and limited olfactory and auditory stimuli [7,9,10]. In particular, the use of heart rate recordings as a non-invasive physiological measure has been repeatedly and reliably used to measure the reaction of horses when exposed to a stressor [5,7,11,12]. However, there is currently little knowledge of the effect of multiple types of auditory stimuli on horses. Existing evidence for differential responses to auditory stimuli in horses remains conflicting. A novel auditory stimulus has been shown to elicit both a behavioural and physiological response [7] and when exposed to a stressful environment, classical music appears to decrease the physiological stress response. However, during isolation, different types of music appear to have little effect on equine behaviours under separation conditions [13]. In addition, the discrimination of stimuli has been utilised in horses to assess the limits of equine sensory perception, including auditory perception, demonstrating an ability to learn to differentiate sounds [14]. Improving knowledge of responses to multiple auditory stimuli, particularly to sounds encountered in yard and competition environments, could enhance our ability to manage horses and improve safety and competition performance.

Sport horses are exposed to a variety of sounds at competitions and often must perform consistently without visibly responding to auditory stimuli. Auditory stimuli can be used to cue the attention of horses and act as a distractor which, unlike visual stimuli, can be perceived during almost any activity [15]. Noise-reducing ear covers (also known as ‘noise cancelling’, ‘sound damping’ ears, or ‘acoustic ears’) have been developed and are commercially available for use on sport horses with the aim of improving focus and attention on the rider and optimising performance. These are permitted in international competition, including for noise-cancelling purposes [16]. However, the effectiveness and mechanism by which this noise reduction is achieved are not fully understood. An assessment of the potentially differential effects these ear covers may have on responses to sounds presented in competition and yard environments could help to optimise the use of ear covers to reduce stress responses and improve the welfare of horses.

To the knowledge of the authors, there are currently no reports of the effectiveness of ear covers in altering the responses of horses to different sounds despite having implications on both horse welfare and sport performance. To address this, the present study aimed to (i) investigate the heart rate and behavioural responses of horses to different sounds commonly present in a competition environment, and (ii) compare these behavioural and physiological responses in horses wearing commercial noise-reducing ear covers and not wearing ear covers to test their effectiveness at reducing noise. It was hypothesised that (i) there would be differences in both the behavioural and physiological responses to different sounds presented, and (ii) there would be a significant difference between the responses in the presence and absence of noise damping ear covers.

## 2. Materials and Methods

### 2.1. Animals

Horses with a range of competition experience were recruited from three competition yards. Horses were included on the basis of familiarity with wearing ear covers either when ridden or in the field, having a normal veterinary clinical examination and an absence of any veterinary history of hearing problems. Eighteen horses met the inclusion criteria (Mean Age = 12.3 years, SD = 5.7 years) and were included in the analyses (Table 1).

Ethical approval was attained for this study (Cambridge University Department of Veterinary Medicine project number CR591) and consent forms were completed by owners/agents of all participants.

### 2.2. Experimental Design

#### 2.2.1. Test Environment

**Figure 1:**
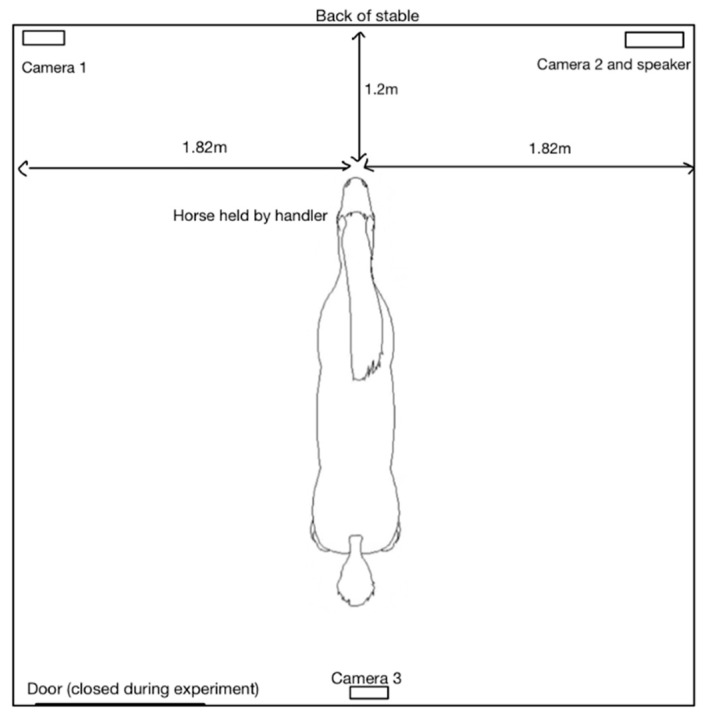
Setup of test stable (3.65m × 3.65m).

A standardised test stable (dimensions 3.65 m × 3.65 m) was set in each location. A speaker was positioned in the corner to the front-right of the horse at a height of 1.76 m. Horses were positioned facing the back wall of the stable to limit any changes in visual stimuli, located 1.2 m from the back wall and 1.82 m from each of the side walls. Each horse wore a headcollar and was held loosely by an experienced and familiar handler (Figure 1). The background noise detected in the test stable was limited as far as possible and background noise levels were recorded in the range of 33–54 dB across all locations. If there was any obvious increase in background noise, no auditory test stimuli were presented to the horses.

#### 2.2.2. Auditory Stimuli

**Figure 2:**
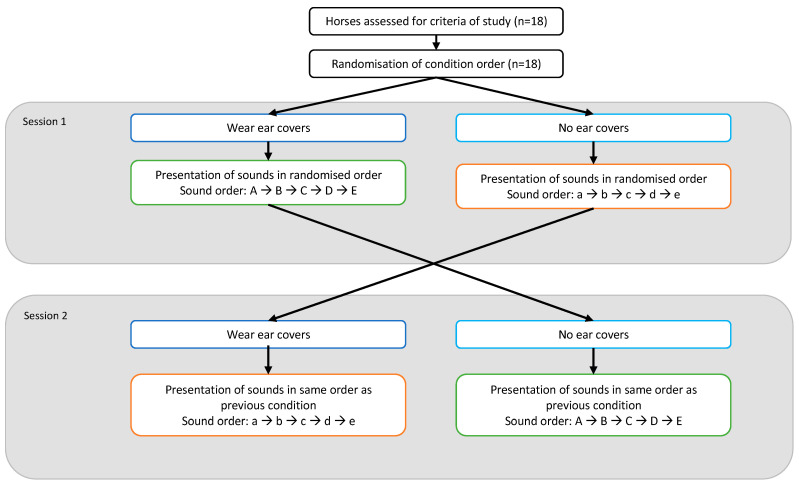
Flow diagram showing the randomised order crossover design used when presenting sounds and comparing wearing and not wearing ear covers. Uppercase and lowercase sets of letters represent different, randomised orders of sounds.

Five different auditory stimuli were presented to the horses (67 dBA), summarised in Table 2. Each sound was played from an Anker SoundCore A3102 speaker (Anker, Changsha, China) for a period of 20 s; a duration based on pilot testing with 5 horses that showed periods of 20 s consistently elicited both behavioural and physiological responses. Horse responses to auditory stimuli were tested with and without noise-damping ear covers. Auditory stimuli were presented to horses in the same order within horses between the conditions of no ear covers (headcollar only) and noise-damping ear covers. To compare responses to different sound types, sounds were presented in a randomised order between horses to take account of any effects of habituation between sounds within a session (Figure 2).

**Figure 3:**
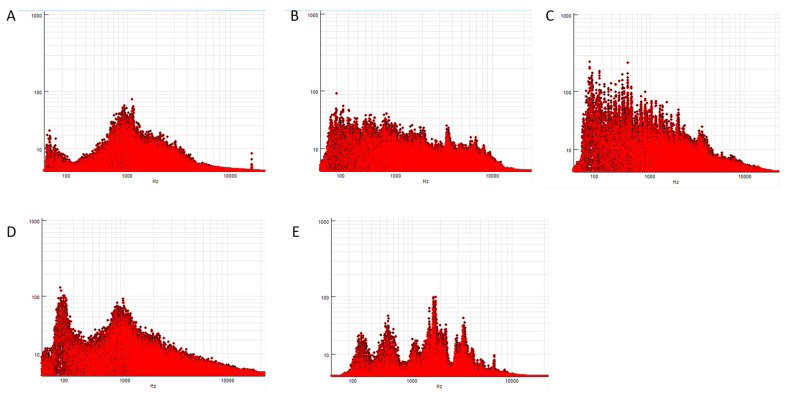
FFT analysis characterising the frequency intensities of the auditory stimuli. (**A**) Applause (two peaks at approximately 2 Hz and 1000 Hz); (**B**) Feed (no distinguishable peaks); (**C**) Music (no distinguishable peaks); (**D**) Trotting (peaks at approximately 100 Hz and 1000 Hz); (**E**) Whinny (peaks at 193 Hz, 513 Hz, 1054 Hz, 3488 Hz, and 6871 Hz).

Fast Fourier Transform (FFT) with a segment length of 2048 (2^11^) samples was applied to 20 s samples of the audio .WAV files to characterise the frequency content of the different sounds using Sigview V6.0.0 (SignalLab e.K., Karl-Abt Straße 5, 75,173 Pforzheim, Germany) (Figure 3).

#### 2.2.3. Noise-Damping Ear Covers

The ear covers comprised a cotton mesh headpiece and 3 layers of material covering the ears (inner and outer layer of cotton and a middle layer of ‘technical soundproof material’). The ear covers are commercially available for use in competition, marketed to reduce response to noise (Gerald Soundless Ears; Equiline, Veneto, Italy). All horses were used to wearing ear covers either when ridden or in the field. The ear covers were fitted on the horse so that the entire ear was covered by the ‘soundproofing’ section of material and an elastic strap under the horse’s mandible was used to secure the ear covers throughout the experiment.

#### 2.2.4. Recording and Analysis of Behaviours

Three cameras (EX-ZR200; CASIO, Tokyo, Japan; 1920 × 1080 pixels; 30 frames/second) were used to record the behavioural responses of the horses, providing continuous simultaneous video of the left and right sides and the caudal aspect of the horse (Figure 1). Each camera was positioned at a height of 1.76 m. The cameras recorded the entire duration that the horse was positioned in the test stable, and the times that sounds were played were recorded.

Videos were downloaded onto a PC and reviewed by a single trained operator for the presence of behaviours using an ethogram (Table 3) adapted from previous studies and based on pilot information. The behaviours were chosen using ad hoc observations of 4 horses in the 2 weeks prior to the experiment taking place and literature on behavioural signals of horses in both stressful and non-stressful environments [7,9,10,12,17,18,19]. Behaviours were recorded during the 20 s period of auditory stimulation to determine the immediate response to each auditory stimulus. Intra-operator repeatability was confirmed by undertaking 4 repeated randomised order analyses of videos of 3 horses chosen at random. The recorded presence of behaviours was identical in all repeats in all of the subjects, confirming intra-operator repeatability.

#### 2.2.5. Heart Rate Monitoring

Heart rate (HR) was recorded using a Polar Equine Heart Rate Monitor (Polar Electro (UK) Ltd., Coventry, UK) comprised of an electrode belt and H10 heart rate sensor, which continuously transmitted heart rate data to a wristwatch throughout the experiment where they were stored and later downloaded onto a PC. Water was applied to the horses’ coats to improve the contact between the electrode areas and the horses’ skin and enhance the heart rate signal quality. The horses were habituated to the heart rate monitoring strap prior to the experiment.

#### 2.2.6. Procedure

The HR monitoring strap was attached to the horse in their own stable or outside the test stable for approximately 2 min to record the resting HR for each horse. The horse was then led into the test stable and was allowed to acclimatise for approximately 2 min without any auditory stimuli presented. Once the HR had fallen to within 2 bpm of the resting heart rate for a period of approximately 15 s, the first sound was played from the speaker. For each auditory stimulus, the average and maximum HR for each horse was determined for the 20 s stimulus period plus the 45 s that followed as observations of the pilot data showed that a period of 45 s after the auditory stimulus ceased was optimal to contain the curve showing exponential decay in HR response to the sound. The duration of increased HR response was recorded for each sound.

A period of at least 2 min was left between the presentation of each sound. Each subsequent sound was not played until the HR had fallen to within 5 bpm of the resting HR for approximately 15 s. Following the presentation of all sounds, the horse was held until the heart rate returned to resting value for 60 s to ensure the resting heart rate was consistent across the entire experiment. The horse was subsequently returned to their own stable and rested for at least 4 h; this period was based on pilot testing in 4 horses, which confirmed an absence of habituation between sessions.

Following the initial data collection, the experiment was repeated for a second session, with or without ear covers in a randomised order, balanced randomised cross-over design. If the horse wore noise-damping ear covers for the first session, they would not be worn for the second session and vice versa.

### 2.3. Statistical Analysis

Data distribution and normality were investigated using a Shapiro–Wilk test. Data were compared within horses between conditions of ear covers and no ear covers and between different auditory stimuli. HR data were not normally distributed so Wilcoxon signed rank and Kruskal–Wallace tests were used. The immediate behavioural responses to auditory stimuli were compared between conditions of ear covers and no ear covers within horses and between different auditory stimuli using a chi-squared (χ^2^) and Fisher’s exact test. Statistical analyses for both heart rate and behavioural data were performed using R 4.0.2 using a significance level of *p < 0.05* throughout. Data are presented in figures as the mean ± standard error mean.

## 3. Results

### 3.1. Responses to Different Auditory Stimuli without Ear Covers

#### 3.1.1. Heart Rate Responses

**Figure 4:**
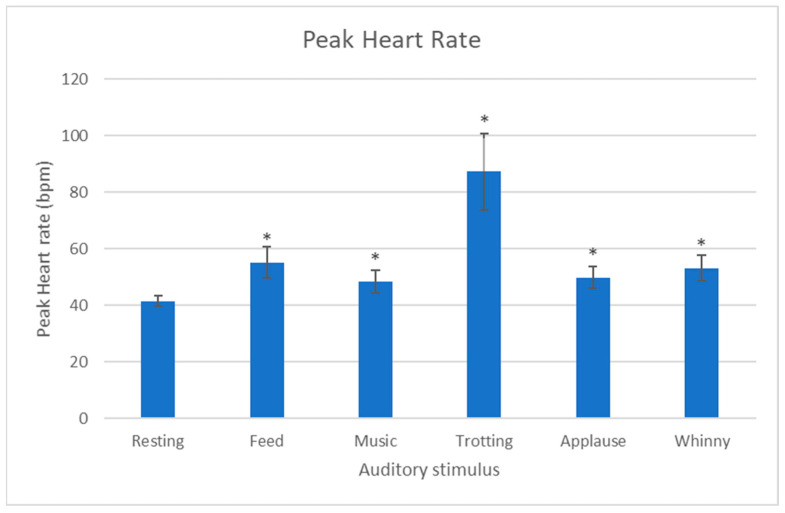
Resting and Peak Heart Rate ± SEM (bpm) of 18 horses in response to five auditory stimuli. * indicates a significant increase in heart rate from resting to peak heart rate.

For every sound, the peak heart rate response was significantly greater than the resting heart rate (Trot: *p* < 0.001, Applause: *p* = 0.003, Music: *p* = 0.003, Whinny: *p* < 0.001, Feed: *p* < 0.001) (Figure 4). A significant difference between sounds was seen for the difference between peak heart rate and resting heart rate (*p* = 0.007). Pairwise comparisons showed significant differences between trotting and all other sounds (trotting-feed: *p* = 0.004, trotting-music: *p* = 0.001, trotting-applause: *p* = 0.002, trotting-whinnying: *p* = 0.002) with trotting eliciting a higher peak HR in each case and also producing the largest peak heart rate of all five sounds (Figure 4).

Similarly, a significant difference between sounds was seen for the increase from resting to mean heart rate response to the auditory stimulus (*p* < 0.001). The mean heart rate in response to each sound was significantly greater than the resting heart rate for trotting (*p* < 0.001), feed (*p* = 0.003), and whinny (*p* = 0.01) sounds.

#### 3.1.2. Behavioural Responses

**Figure 5:**
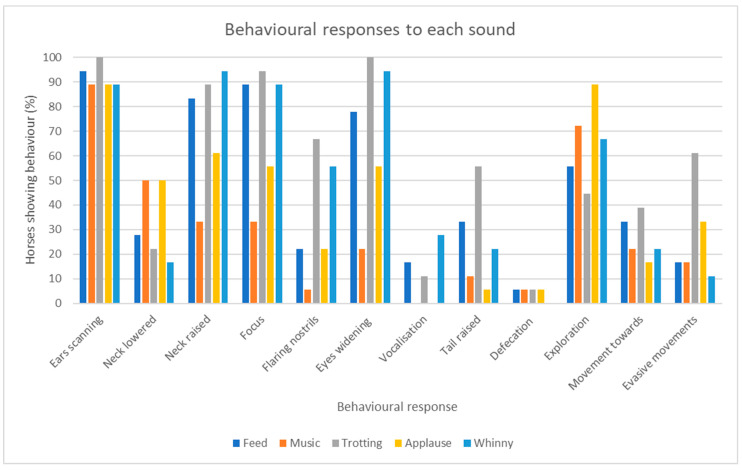
Percentage of horses eliciting behaviour in response to each sound (n = 18).

The behavioural responses to all five sounds are summarised in Figure 5. There was an overall difference between sounds presented in the frequency of behaviours (χ^2^ (44) = 62.0, *p* = 0.038). Ears scanning was a frequent behavioural response to all sounds. The behaviours of evasive movement and raised tail were relatively overrepresented as a response to the trotting stimulus. Exploratory behaviour was relatively underrepresented in response to trotting but overrepresented in response to applause. In addition, vocalisations were frequent in response to whinnying compared to the other sounds and the neck lowered behaviour was frequent in response to music and applause.

### 3.2. Comparing Responses with and without Ear Covers

#### 3.2.1. Heart rate responses

**Figure 6:**
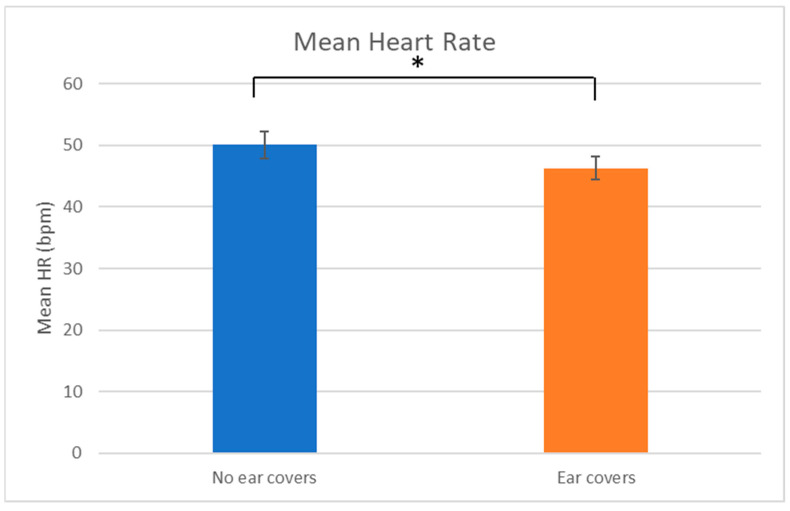
Overall mean heart rate (bpm) ± SEM with and without ear covers. * indicates a significant differences.

The mean HR was significantly different when comparing overall between wearing and not wearing ear covers with mean HR lower when wearing ear covers (No ear covers: 50.1 ± 2.2 bpm, Ear covers: 46.3 ± 1.9 bpm; *V* = 1498, *p* = 0.027; Figure 6). Taking each sound individually, only the feed sound showed a significant difference in mean HR with mean HR higher without ear covers (No ear covers: 47.5 ± 2.8 bpm, Ear covers: 42.1 ± 2.1 bpm; *V* = 36, *p* = 0.030).

**Figure 7:**
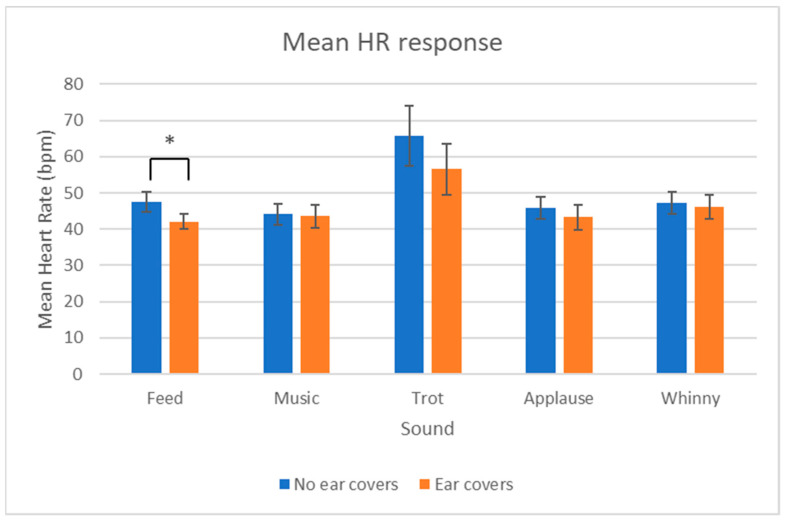
Mean heart rate ± SEM with and without ear covers for each of the sounds. * indicates a significant differences.

The increase in HR from resting to peak was also compared with and without ear covers and it was found that the increase was only significantly different between the two conditions for the feed sound (*V* = 96, *p* = 0.007; Figure 7) with the increase in HR larger without ear covers.

#### 3.2.2. Behavioural Responses

**Figure 8:**
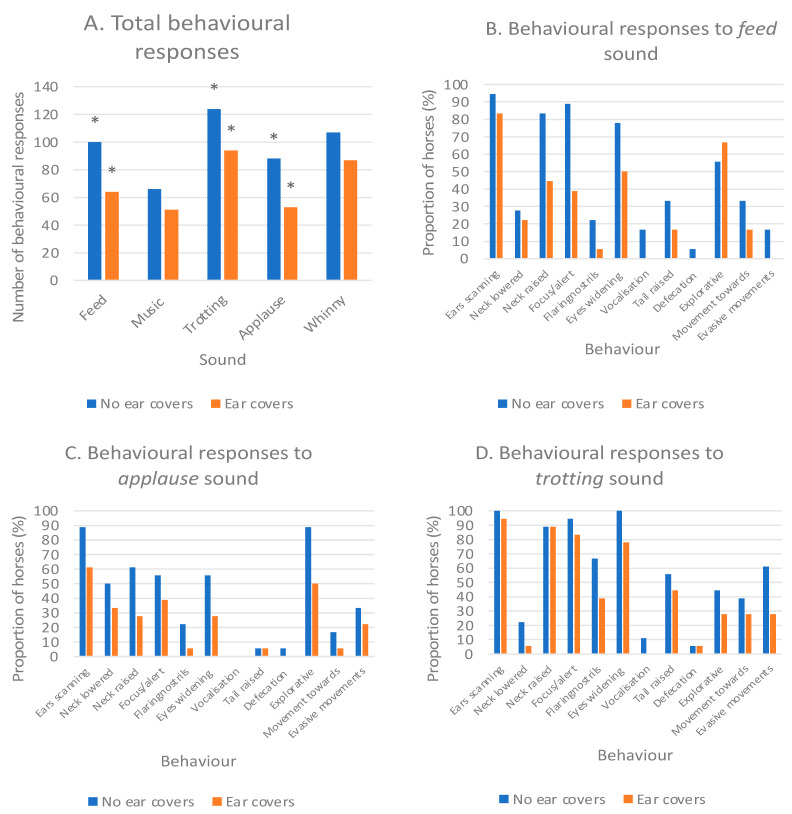
Behavioural responses with and without ear covers: (**A**) Total number of behavioural responses, * indicates a significant difference; (**B**–**D**) Behaviours with and without ears in response to the sounds producing significant differences: (**B**) feed, (**C**) applause, and (**D**) trotting.

There was an overall difference between ear covers and no ear covers for the presence of behavioural responses when taking into account all sounds (*p* < 0.001), with fewer behaviours elicited when wearing ear covers. The total frequency of behaviours for each sound between the two conditions was compared and significant differences between ear covers and no ear covers were seen for trotting (*p* = 0.005), applause (*p* = 0.005), and feed (*p* < 0.001) with no ear covers producing more behavioural responses for all sounds (Figure 8).

## 4. Discussion

This study demonstrated that (i) there were differences in both physiological (heart rate) and behavioural responses to different complex auditory stimuli commonly heard in stable and competition environments, (ii) there was an overall difference in physiological and behavioural responses with and without ear covers, although the only difference in heart rate between ear covers and no ear covers that individually achieved significance was the feed sound.

### 4.1. Responses to Different Auditory Stimuli

Increased HR for all sounds and an overall difference in behavioural responses showed both the physiological and potentially emotional response to a variety of auditory stimuli. The overall responsiveness to the complex auditory stimuli suggests that noise could have an impact on the focus and attention of horses during competition, potentially distracting the horses and producing a sub-optimal performance [15]. This may be useful in understanding the importance of auditory stimuli, and relevant to considering the set-up of yard and competition venues to improve horse relaxation and performance.

Many behavioural responses could be attributed to the attempt to localise and attend to the sound given the lack of visual stimuli. For example, ‘ears scanning’ was high for all sounds, possibly as a way of gaining sensory information in order to attend to the source of the stimulus. Similarly, increased ‘exploration’ in response to applause could be an extension of this as a way to visually localise and establish the identity of the sound so that the horse can respond more appropriately using combined visual and auditory information [21]. The decreased ‘exploration’ response in combination with increased ‘tail raised’ and ‘evasive movements’ when exposed to trotting could indicate an innate flight response due to the perception of a potential threat. In contrast, the increased proportion of ‘neck lowered’ behaviour in response to music could suggest a calming effect of music. This was noted in conversations with some riders who commented on the calming effect they perceived to take place when music was playing quietly while riding. In support of this, classical music has been shown to reduce the intensity of stress responses when exposed to a stressful situation [22]. Perception and, by extension, resulting behaviour, is driven by both bottom-up (sensory input) and top-down (cognition and experience) processing so inter-individual responses to sounds only experienced in a domestic or sporting environment, including music and applause, are likely to depend on the prior experience of the individual horse [23]. The effect of music may therefore depend more on the intensity of the stimulus, the individual experience of the horse and the environment in which it is played rather than the stimulus itself. Further investigation of this may contribute to the improvement of horse welfare in competition and award ceremonies in all equestrian disciplines by informing competition organisers.

Increased vocalisations in response to the whinny sound could be a demonstration of the role of vocalisations in animal communication, particularly in a stressful situation [24]. It is worth noting that stress can be either eustress (positive) or distress (negative) and the frequencies of whinnies produced in response to each type of stress have been shown to be different [25] so the properties of the specific whinny stimulus used could be influential in the resulting behavioural and physiological response. In addition, horses appear to cross-modally recognise the familiarity of the caller, integrating auditory and visual information to respond accordingly [26,27]. It is unclear from the FFT graph alone whether the whinny implies distress or eustress; however, the increased proportion of stress responses elicited by the whinny sound such as ‘neck raised’, ‘flaring nostrils’, and ‘eyes widening’ could indicate a situation of distress due to the call of an unknown horse that can be heard but not seen (a mismatch of perceptual cues). In a competition environment, this may occur in an indoor arena for example where a call of an unknown horse outside the arena can be heard but not seen. The results recorded in this study could therefore allow riders to better predict the response of their horse when exposed to the whinny of an unknown horse.

In both the HR and behavioural results, trotting produced the largest response. This may be due to alarm in response to the perception of a threat. Horses are social animals and have evolved to live in herds so may respond to trotting by acting in a similar way through behaviours such as evasive movement accompanied by an elevated HR response as part of an innate flight response, which although reduced in domesticated horses, still may persist [28].

### 4.2. Effect of Wearing Ear Covers

An overall decrease in behavioural and heart rate responses with ear covers on suggests that the ear covers do have an impact on reducing noise detection by horses. Therefore, wearing ear covers could potentially reduce stress, or improve the focus and attention of a horse on its rider in a training, competition, or prize-giving environment. While it may be optimal to remove potential sound stressors from a horse’s environment, this may be difficult to achieve in an uncontrolled environment, where there could be a potential benefit to using ear covers to reduce stress.

Interestingly, when examining responses to individual sounds, the ear covers had no effect for the whinny sound. This may be because horses are likely to have high sensitivity to the frequency range of whinnies as vocalisations are central to equine communication of emotional and physiological states [29]. Therefore, the presence of noise-damping ears may have a smaller relative effect when reducing the detection of this type of stimulus. This could be significant for sport horses that compete in an arena that is close to a warm-up containing other horses that may call during a performance and distract the competitor. Since different whinnies can infer different states of stress [25], further studies would need to be conducted with a range of whinnies with differential peaks of frequency amplitude to fully understand the impact of ear covers on the perception and response to different types of vocalisations.

Although, overall, both HR and the total number of behavioural responses were larger when not wearing ear covers, there were inconsistencies between the HR and behavioural responses when comparing individual sounds. Differences in behaviour were seen for feed, trotting, and applause whereas a significant difference in HR was only seen in response to feed. Physiological and behavioural signals have been shown to correlate and have a close relationship when examining stress responses [10]. However, evidence is mixed for different types of stimuli and past studies have shown that physiological and behavioural signals are not always consistent [7,10,30]. In this case, the responses may not be matched due to a reduced ability to localise sound with ear covers on. It has been suggested that a ‘fear state’ is caused by the identification of an actual danger, whereas an ‘anxiety state’ is caused by a potential danger that cannot be fully localised or identified [8]. In the case of trotting and applause, the horse may be placed in a distress situation. The presence of ear covers could result in fewer behavioural responses despite no reduction in heart rate because the horse may be preparing for a generalised, adaptive flight response to danger but is unable to localise and identify the sound as well so does not elicit the appropriate behavioural response. The ability to localise the sound could not be directly tested in this study as the sound was only presented from one direction so further studies could investigate this by presenting the sounds from different locations in the test stable. Thus, a generalised autonomic response through the physiological system could be occurring during a competitive performance without visible behavioural responses when wearing ear covers. Three stages of behavioural and physiological responses have been previously identified for a novel visual stimulus [10]. Although the specific chronology of behavioural and physiological responses was not measured in this study, observations from the behavioural and physiological analysis indicate that responses followed a similar pattern. Ear scanning was typically the first behaviour to be expressed, indicating stimulus recognition and evaluation followed by the addition of further behavioural responses and an increase in heart rate, which could indicate a moderate fear state and, in some cases, a flight response.

In addition, the mechanism by which the ear covers could be reducing noise transmission should be considered, despite not being directly tested in this study. Although the material itself is marketed as ‘soundproofing’, we postulate that additional noise produced from interaction between the ear cover material and ear hairs themselves could also have an impact on the perception of external stimuli, particularly during ‘ear scanning’ when there is additional movement between the ears and covers. Auditory stimuli can cue the attention of horses so a consistent noise close to the ear may distract from external signals and enable greater focus in a competition environment. The frequencies that are transmitted through the ear cover material should also be considered in this mechanism. Horses are known to detect complex sounds with high-frequency components with greater acuity than humans [31] so if ear covers are successful in blocking high-frequency components of sounds, the predictability of response by the rider may be increased. However, the results of this study indicate ear covers reduced responses to sounds with amplitude peaks at lower or distributed frequencies which would suggest that the ear covers may be blocking lower frequencies more consistently. As prey animals, horses are adapted to have high-frequency hearing so, unlike other large mammals, have poor acuity at lower frequencies [23]. This could support a suggestion that noise potentially produced from the movement of the ear cover material against the ear itself could be competing with this low-frequency external stimuli, further contributing to the lack of perceptual acuity at lower frequencies. Further characterisation of the mechanistic properties of ear covers needs to be established to confirm this.

### 4.3. Limitations

Due to the experimental setup in a test stable, it cannot be directly determined from this study how the horses would react in a competition environment. In ‘real-world’ environments, such as competition arenas, sound is usually perceived in association with visual and potentially olfactory stimuli and may be presented in combination with other noises from differing directions. Exposure to these additional stimuli in an unfamiliar environment in such scenarios may lead to stacking of stressors. These factors may heighten a horse’s awareness and alter their response to sound stimuli. In addition, most equestrian sports involve a rider on the horse. The collective response of both horse and rider could alter the effectiveness of ear covers. Further studies in competition settings or in ridden horses would further knowledge in this area and provide valuable information on sport horse welfare. Although this study, like previous studies [7,10,12], used heart rate as an outcome measure, further information on stress response in different environments could also potentially be investigated using heart rate variability.

Habituation has been shown to occur in response to visual stimuli when assessing fear responses [10]. This study aimed to reduce the potential impact of this effect through a randomised order and balanced crossover design and was informed by pilot studies to dictate the time left between test sessions. Future studies could test the effects of habituation over a longer period of time using these sounds to investigate any differences in habituation to each type of sound. This could have implications in sports as increased exposure to a sound may not only reduce responses to the sound but also potentially improve the effectiveness of wearing ear covers.

## 5. Conclusions

To summarise, this study shows that horses show different behavioural and physiological responses when presented with different auditory stimuli, suggesting an ability to discriminate the sounds and alter responses based on the individual stimulus. When wearing noise-damping ear covers, these responses are attenuated implying a reduction in the perception of the auditory stimuli with varying levels of effectiveness for different sounds.

## Figures and Tables

**Table 1:** Overview of participants.

Horse	Age	Breed	Sex	Competition Experience (Level, Discipline)
1	19	Warmblood	Gelding	International dressage
2	15	Warmblood	Gelding	International dressage
3	8	Warmblood	Gelding	Local dressage
4	19	Warmblood	Gelding	International dressage
5	13	Warmblood	Gelding	International dressage
6	4	Warmblood	Gelding	None
7	22	Cob	Mare	None
8	3	Warmblood	Stallion	None
9	4	Warmblood	Mare	Local dressage
10	6	Warmblood	Gelding	National dressage
11	17	Native pony	Gelding	Local multiple disciplines
12	14	Cob cross	Gelding	Local dressage, showjumping, hunter
13	12	Thoroughbred	Gelding	Retired national hunt
14	15	Cob	Gelding	National showing; local dressage and showjumping
15	11	Thoroughbred	Gelding	Local eventing
16	15	Warmblood	Gelding	International showjumping; local dressage
17	8	Thoroughbred cross	Gelding	Local multiple disciplines
18	17	Native pony	Mare	National level showing; local showjumping, cross-country, and dressage

**Table 2:** Auditory stimuli presented for 20 s at an intensity of 67 dBA at the horse’s head.

Sound	Description
Whinny	Whinny of an unknown horse was repeated 4 times within the 20 s period
Applause	Crowd clapping and cheering
Trotting	Sound of hooves on hard surface in a 2-beat gait
Music	‘We are the Champions’ by Queen, commonly used in competition prize-giving ceremonies
Feed	Horse nuts being scooped into a rubber feed bowl

**Table 3:** Equine Ethogram.

Behaviour	Description
Ears scanning	Ears flick back and forth at varying speeds [9].
Neck lowered	Neck lowered so the horse’s eyes are below the height of the withers [9].
Neck raised	Neck raised so horse’s eyes are above the height of the withers [9].
Focus/alert	Head turned and held stationary towards auditory stimulus source with ears pricked forwards for at least 3 s [9,20].
Flaring nostrils	Nostrils widen from resting position.
Tail raised	Fleshy part of the tail is lifted to or above horizontal [9]
Vocalisation (whinny)	A high amplitude call of long duration that has a fluctuating frequency.
Eyes widening	Eyes widen showing whites of eyes for more than 3 s.
Defecation	Elimination of faeces.
Exploration	Neck in horizontal position or lower, searching or sniffing different parts of the test stable [18,20].
Movement towards sound	Step in direction towards auditory stimulus.
Evasive movement	Steps in direction away from auditory stimulus.

## Data Availability

The data presented in this study are available on request from the corresponding author.
